# Impact of Dietary Palmitic Acid on Lipid Metabolism

**DOI:** 10.3389/fnut.2022.861664

**Published:** 2022-03-23

**Authors:** Elisabetta Murru, Claudia Manca, Gianfranca Carta, Sebastiano Banni

**Affiliations:** Department of Biomedical Sciences, University of Cagliari, Cagliari, Italy

**Keywords:** palmitic acid, *de novo* lipogenesis, fatty acid metabolism, dietary fatty acids, saturated/unsaturated ratio

## Abstract

Palmitic acid (PA) is ubiquitously present in dietary fat guaranteeing an average intake of about 20 g/d. The relative high requirement and relative content in the human body, which accounts for 20–30% of total fatty acids (FAs), is justified by its relevant nutritional role. In particular physiological conditions, such as in the fetal stage or in the developing brain, the respectively inefficient placental and brain blood–barrier transfer of PA strongly induces its endogenous biosynthesis from glucose *via de novo* lipogenesis (DNL) to secure a tight homeostatic control of PA tissue concentration required to exert its multiple physiological activities. However, pathophysiological conditions (insulin resistance) are characterized by a sustained DNL in the liver and aimed at preventing the excess accumulation of glucose, which result in increased tissue content of PA and disrupted homeostatic control of its tissue concentration. This leads to an overaccumulation of tissue PA, which results in dyslipidemia, increased ectopic fat accumulation, and inflammatory tone *via* toll-like receptor 4. Any change in dietary saturated FAs (SFAs) usually reflects a complementary change in polyunsaturated FA (PUFA) intake. Since PUFA particularly n-3 highly PUFA, suppress lipogenic gene expression, their reduction in intake rather than excess of dietary SFA may promote endogenous PA production *via* DNL. Thereby, the increase in tissue PA and its deleterious consequences from dysregulated DNL can be mistakenly attributed to dietary intake of PA.

## Introduction

Palmitic acid (PA) is one of the most abundant saturated fatty acids (SFAs) in nature, which is present in animal and human tissues, plants, algae, fungus, yeast, and bacteria. Its distribution varies both within species and among species, and its content can be influenced by several environmental factors as the variation of soil pH, nutrient–ion interaction, age, water, and climate (1, 2).

The average dietary intake of PA is around 20–30 g/d representing about 8–10%en (3–5) and can be found in different vegetable and animal fat sources (Table 1) (6), with levels of 20–30% in animal lipids and 10–45% in vegetable oils. Methods that are used to prepare the food also impact on PA amount; for example, in processed and preserved meats, the content is higher than fresh meat with values up to 7.6/100 g of edible portion in salami and in lard 21/100 g of edible portion. It should be pointed out that due to high within-food variability of PA content, it is very difficult to assess its precise dietary intake. In addition, PA absorption and metabolic fate are strongly influenced by several factors, such as food matrix and pathological or physiological conditions.

**Table 1:** Palmitic acid content of oils and fats from vegetable and animal sources (expressed as percentage mass fraction of total FAs) (6).

**Vegetable sources**	**% Fraction/total FA**	**Animal sources**	**% Fraction/total FA**
Palm oil	40.1–47.5	Lard	21.07
Cottonseed oil	21.4–26.4	Goose	7.41
Cocoa butter	25.4	Whole chicken	2.19
Olive oil	7.5–20.0	Pork loin	2.06
Oat bran oil	17.4	Lamb	0.58–1.99
Avocado oil	17.2	Rabbit	1.22–1.95
Wheat germ oil	16.6	Beef meat	0.31–1.14
Corn oil	8.6–16.5	Horse meat	1.65
Peanut oil	8.3–14.0	Sheep meat	0.58
Soya bean oil	8.0–13.3	Goat meat	0.40
Grapeseed oil	5.5–11.0	Deer meat	0.12
Sesame oil	7.9–10.2	Salami	5.73–7.55
Coconut oil	8.2	Mortadella	5.70
Walnut oil	3.9–7.2	Ham	3.93–4.93
Linseed oil	4.0–7.0	Speck	3.71
Almond oil	6.5	Pancetta	5.67–5.99
Safflower oil	4.8–6.2	Butter	20.86
Linola oil	6.0	Parmesan cheese	8.04
Cashew nut oil	4.0–6.0	Fontina cheese	7.31
Rapeseed oil	1.5–6.0	Cream	5.72
Sunflower oil	5.4–5.9	Ricotta cheese (cow)	3.49
Hazelnut oil	5.2	Ricotta cheese (sheep)	2.85
Canola oil	4.0	Cow's whole milk	0.92–1.97
Eggs sources		Sheep's whole milk	1.58
Hen egg (whole)	1.90–5.90	Goat's whole milk	1.34
Duck egg	3.00	Semiskimmed milk	0.45
Turkey egg	2.72	Yogurt	0.92

## Importance of the Matrix and PA Distribution on Metabolism

In evaluating the effects of food on health, the overall macronutrient composition and structure need to be considered, i.e. the “food matrix” (7), meaning that food chemical compounds behave differently in isolated form in comparison to part of food structures (8), as well as the resistance of a food to the mastication and the viscosity of aliments, which may affect the bioavailability and digestibility of dietary lipids (9). Dietary fats comprise cholesterol and fatty acids (FAs), which can be free or components of complex lipids, as triacylglycerols (TAGs), and phospholipids (PLs), organized in structures able to modulate FA final metabolic fate. FA esterification on different positions of the TAG glycerol backbone (central sn-2 position, external sn-1 and sn-3 positions) or on a PL may also impact on their digestibility and metabolism (10–16). PA in foods is mainly present esterified in PL and TAG.

Dietary PL represents 1–10% of total daily fat intake (17). PL is mainly catabolized by pancreatic phospholipase A2 (PLA2) that produces free FA (FFA) and lysophosphatidylcholine (lysoPL), which once absorbed by the intestinal epithelium are reacylated or hydrolyzed to form PL or glycerol-3-phosphorylcholine, respectively. FFAs are instead used for TAG synthesis that are subsequently incorporated into chylomicrons (17).

Over 90% of dietary FAs are esterified to TAG preferentially hydrolyzed by digestive lipases (18) on sn-1,3 positions followed by pancreatic lipase to give 2-monoacylglycerol (2-MAG) and FFA (15), which cross the apical membranes of the enterocytes and are reassembled into TAG for secretion to plasma in chylomicrons. SFA released from positions sn-1 and sn-3 may form insoluble soaps with ions as calcium that are not absorbed, a singularity lost if SFA is in the TAG sn-2 position (19), as confirmed by animal and human infant studies which demonstrate that sn-2 esterified FA is efficiently absorbed as 2-MAG (20, 21). The peculiar sn-2 position of PA in human milk results from the activity of the glycerol-3-phosphate (G-3-P) acyltransferase, present in the mammary gland, which acylates an unsaturated FA at the sn-1 position of G-3-P and subsequently a PA at the sn-2 position (22, 23). Human milk, which contains 20–25% of PA with respect to the total FA whose 70% is in sn-2 of TAG, limits PA malabsorption providing the infant with high PA (19, 23–25). Conversely, 45 and 58% of cow and rodent milk fat (25 and 15% of PA on the total FA, respectively) are esterified at the TAG sn-2 position (23, 26). FA composition of the early diet influences intestinal membrane FA, which affects nutrient transport, permeability, and inflammatory pathways that persist into later life (27, 28). Notably, PA also plays an important role in the developing fetus with the term infant reaching 13–15% of body fat of which 45–50% is PA, mostly derived from endogenous synthesis in the fetus (29).

PA peculiar tissue distribution results in its better incorporation in several tissues, for example, adipose tissues, with a lower deposition of fat in the visceral depots and higher in the subcutaneous fat (30). Interestingly, several studies demonstrated the protective effect of breastfeeding against obesity in childhood (31, 32) and adulthood (33–36). Also, donkey milk contains high concentrations of PA in sn-2 of TAG and is recognized as the best potential substitute for human milk due to its remarkable nutritional value, good palatability, and reduced allergenicity (37, 38). A recent study in rats showed that oral supplementation with human or donkey milk ameliorated metabolism and reduced inflammation potentially mediated by an improved redox status, mitochondrial uncoupling, and dynamics (39). In addition, it has been demonstrated that PA in sn-2 by modifying endocannabinoids and congeners biosynthesis in different tissues may potentially concur in the physiological regulation of energy metabolism, brain function, and body fat distribution (40). In contrast to milk, in animal tissues that include human adipose tissue and also beef tallow and in soybean oil and cocoa butter, PA is mainly at sn-1,3 position, whereas the sn-2 is occupied by an unsaturated FA (41–45). Lard, having high amounts of PA at TAG sn-2, represents an exception (23), and in animal studies, PA from lard was better absorbed with respect to PA from cocoa butter and palm oil (46, 47). This PA peculiar position led food industries to often use interesterification to produce functional infant formula containing TAG with a high amount of sn-2 PA (48, 49). Amounts of PA in the sn-2 position in breast-fed infants (81%) or in infants fed formula prepared with synthesized TAG (39%), plasma chylomicron TAG containing PA in sn-2 position were higher with respect to those fed with standard infant formula with 6% of PA in sn-2 position (50), and it was shown in infants that PA loss in stools was 8-folds less using infant formula with lard TAG with respect to randomized lard (51). Also, an increase in the proportion of sn-2 PA by interesterification of TAG in coconut oil and palm olein improved PA absorption and metabolism in rats (52, 53). Therefore, the matrix/esterified position plays a crucial role in determining the metabolic fate of dietary PA.

## Assessment of Dietary PA Intake in Humans

Most of the studies aimed at evaluating dietary FA intake rely on food frequency questionnaires (FFQs), and food diaries where even repeated measurements do not necessarily provide valid measures of individual intake. Extreme intakes may reflect under- and overreporting rather than true low or high intakes, and subjects most prone to reporting bias may be repeatedly misclassified in quantiles of the distribution (54). In addition, assessing the precise nutrient intake is quite difficult because of the errors made in recalling or the identification of the amounts of foods eaten, especially in processed foods (55).

Measurement of circulating PA is not also a reliable marker of its dietary intake; in fact, the dietary consumption of PA has low impact on plasma levels compared with its endogenous biosynthesis; data from a controlled human feeding trial showed that variations in SFA intake from 11 to 30%en did not change circulating SFA, including PA (56). Accordingly, cohort studies did not show a solid correlation between the PA dietary intake (evaluated by FFQ) and its plasma levels (*r* = −0.02 to 0.09) (57–60).

Factors other than dietary intake have been suggested to influence FA composition in tissues, first FA metabolism efficiency, genetic variations, and even intrauterine and perinatal program. In fact, considering the relationship between the tissue FA composition and dietary fat, among plasma lipid fractions, only TAGs appear to reflect dietary polyunsaturated FA (PUFA) and SFA, but not monounsaturated FA (MUFA) (61) within the first hours after intake (62). Whereas, FA in serum cholesteryl esters (CEs) and in PL is related to average intake of dietary FA composition during the previous 3–6 weeks, FA of erythrocyte membrane PL and adipose tissue TAG reflect the dietary fat intake of previous months or years, respectively (62).

Noteworthy, it has been demonstrated, by isotope labeling studies in men, that low-fat high-carbohydrate diet stimulates *de novo* lipogenesis (DNL) with the accumulation of VLDL-TAG PA that led to linoleic acid (LA) reduction probably due to dilution effect, whereas with high-fat (40% fat, 45% carbohydrate) DNL is neglectable (63). This suggests that circulating PA levels are largely driven by endogenous synthesis through DNL rather than direct dietary intake. Therefore, the relative strict regulation of PA tissue concentration, with variable amount of the endogenously produced, leads to a high unreliability of the use of PA plasma levels as a tool to determine its dietary intake.

The potential increase of tissue PA by dietary intake is prevented by the contribution of its conversion to palmitoleic (POA), by the insertion of one double-bond through stearoyl-CoA desaturase-1 (SCD1) (62), which reduces PA availability in tissues, but also *via* elongation to stearic acid (SA) and further desaturation *via* SCD1 to form oleic acid (OA). A possible protective capacity of OA to drive PA to be deposited in the neutral form of TAG (64, 65) and POA to improve insulin sensibility has been described (66).

## Fate or Metabolism of PA From *DNL*

When the energetic sources are in excess, the non-fat surplus, mainly carbohydrates, is converted to FA by DNL, a pathway that begins with the conversion of acetyl-CoA into malonyl-CoA by acetyl-CoA carboxylase (ACC). During fed and insulin-stimulated conditions, ACC increases malonyl-CoA levels whereas AMP-activated protein kinase (AMPK) stops the synthesis, probably by inhibiting sterol regulatory element-binding protein (SREBP) (67).

Further evidence indicates that adipose tissue DNL supports metabolic homoeostasis of distant organs, as in liver and muscle, by producing cytokine-like lipids, lipokines, with antidiabetogenic and antiinflammatory activities, such as POA and branched FA esters of hydroxy FA (FAHFA) (66, 68).

In normal conditions, adipose tissue is the major site for DNL, which significantly contributes to body lipid reserves, energy storage, and to the maintenance of serum TAG homeostasis that derived instead from dietary sources (69–75). Furthermore, adipose tissue DNL is considered as an energy-inefficient source of lipids because it yields fewer lipids per calorie consumed, thus being a promising strategy for the treatment of lipotoxicity-related diseases. In fact, adipose tissue DNL is positively correlated with postprandial energy expenditure (76) subsequently to carbohydrate overfeeding, but not fat overfeeding which failed to significantly increase any component of energy expenditure (77, 78).

On the other hand, under specific conditions in the liver, such as insulin resistance, the impaired glycogen biosynthesis and consequent accumulation of glucose induce DNL that may contribute up to 26% to ectopically intrahepatocellular lipids in the pathogenesis of nonalcoholic fatty liver disease (NAFLD). In fact, hepatic DNL is positively correlated with insulin resistance and fatty liver, whereas the correlation with adipose tissue DNL is the opposite (73, 79–81).

In addition, a high-carbohydrate diet, particularly rich in simple sugars as fructose (82–84), activates a lipogenic response and increases the synthesis and secretion of VLDL in liver (85) contributing to hypertriglyceridemia (74). DNL contributes to 10–35% of the total VLDL-TAG pool, probably increasing the size (~130 nm), but not the number of VLDL secreted (86), and is in general higher in insulin-resistant states, and in overweight subjects compared to lean individuals (87–91).

Regulation of DNL occurs through the regulation of transcriptional factors as SREBP-1c and carbohydrate-responsive element-binding protein (ChREBP), activated by increased insulin signaling and increased glucose concentrations, respectively, and both induced by feeding (85, 92–95).

In liver, PUFA downregulates DNL *via* decreased expression of SREBP-1c (96), and leptin reduced adipogenesis through the inhibition of SREBP-1c expression (97). In addition, insulin and SREBP-1c stimulate peroxisome proliferator-activated receptor-γ (PPAR γ) expression (98, 99), which regulates glucose and lipid metabolism thus having adipogenic and lipogenic effects (100) and promotes FA storage in mature adipocytes by the stimulation of lipoprotein lipase (LPL), CD36, and glucose transporter GLUT-4 (101–103).

Therefore, DNL has a dual function, to supply PA in deficiency conditions, such as in the fetus (29) and developing brain (104) to overcome the difficulties of PA to pass, respectively, the placenta and the brain–blood barrier and to prevent the excess accumulation of glucose in the liver. In the latter case, significant increase of tissue PA is detected eluding the homeostatic control of tissue PA concentration and increased endogenous PA production may enhance inflammatory susceptibility through toll-like receptor (TLR4) activation (105) and insulin resistance by ceramide accumulation (106).

## Considerations Over High SFA Diets vs. PUFA-Deficient Diets on Metabolism

Dietary guidelines recommend limiting SFA intake to <10% of calories per day. Correlation between dietary SFA intake and cardiovascular disease (CVD) is quite controversial (107). The Cochrane analysis showed an association between reducing SFA intake and a reduction in cardiovascular events and replacing the energy from SFA with PUFA appear to be useful strategies, whereas effects of replacement with MUFA are unclear (108). SFA increases LDL plasma particle concentration but also their size, which is less associated with CVD (109) because more rapidly cleared than small-dense LDL particles from the circulation due to reduced receptor-mediated uptake (110). SFA increases blood total, LDL, and HDL cholesterol concentrations and decreases fasting TAG concentrations not changing the total–HDL cholesterol (TC/HDL) ratio. The capacity of increasing circulating HDL levels decreases with increasing chain length of SFA and for some studies, but not all, myristic acid and PA, and also carbohydrate intake, negatively affect TC/HDL ratio (111).

*In vitro* cell culture studies showed that PA in the free form in the medium elicits, insulin resistance (112), inflammation *via* TLR4 (105) and prometastatic activities (113), which implies that increased dietary PA may result in higher PA availability to cell tissues in the free form, while as already mentioned, higher intake of SFA results in a decrease of circulating PA in the free form and increase of its monounsaturated metabolites POA and OA. Therefore, *in vitro* models, while may elucidate a limited molecular mechanism, are by far not mimicking pathophysiological conditions. The extremely high concentrations typically used *in vitro* are not achievable *in vivo*, thus the results obtained do not prove any relevant pathophysiological information.

Any change in dietary SFA reflects a complementary change in MUFA and/or PUFA intake. As mentioned above, PUFA (70), particularly n-3 highly PUFA such as EPA and DHA, suppresses lipogenic gene expression by reducing the nuclear abundance and DNA-binding affinity of transcription factors responsible for imparting insulin and carbohydrate control to lipogenic and glycolytic genes (114); thereby, most of the detrimental effects should be ascribed to the lower PUFA intake rather than high dietary SFA.

From, a meta-analysis of randomized controlled trials emerged that replacing 5% energy from carbohydrate with SFA had no significant effect on fasting glucose but lowered insulin, and replacing SFA with PUFA lowered glucose, HbA1c and HOMA. This suggests that consuming more unsaturated FA in place of either carbohydrates or SFA will help to improve blood glucose control while exchanging dietary carbohydrate with SFA does not appreciably influence markers of blood glucose control, and therefore an approach based only on reducing carbohydrates or SFA intake, without considering the source of energy replacement would not be optimal (115).

Data are often contradictory and may be difficult to interpret into dietary advise: some studies suggested that n-6 PUFA would increase CVD risk (116, 117), and therefore the Institute of Medicine recommends a relatively modest range of 5%–10% energy consumption from PUFA, limiting its plausibility as a meaningful replacement for SFA (118). Increasing dietary PUFA may not be desirable as dietary levels of LA are already higher than recommended (119), particularly the n-6/n-3 PUFA ratio (119). The physiological role played by the SREBP-1c, which is inhibited by n-3 FA (114) and in general by PUFA (18), for glycogen biosynthesis and overall glucose homeostasis (120), stresses the point that balance between different dietary FA is strongly recommended and any unbalance may lead or increase the chance to set into motion a disrupted metabolism. In fact, while replacing SFA with LA has an established cholesterol lowering effect, it has not been shown that this lowering reduces mortality (107).

In addition, recently, it has been shown that lower dietary PUFA/MUFA and n-3/n-6, and not SFA, were associated with disturbances in metabolic syndrome-related indices in postmenopausal women, and that polymorphisms of FA desaturase FADS1 (rs174546) and FADS2 (rs3834458) were associated with unfavorable FA profile in red blood cells (121). It has also been demonstrated that the polymorphism rs1761667 of multifunctional CD36 scavenger receptor that facilitates FA uptake and oxidation, leads to a distinct metabolic pattern in normal weight and in obese subjects (122). Thus, changes of tissue FA profile and associated metabolic changes may also be determined by different genetic polymorphisms, which should be considered in developing personalized therapeutic strategies for ameliorating dyslipidemia and other metabolic disorders.

**Figure 1:**
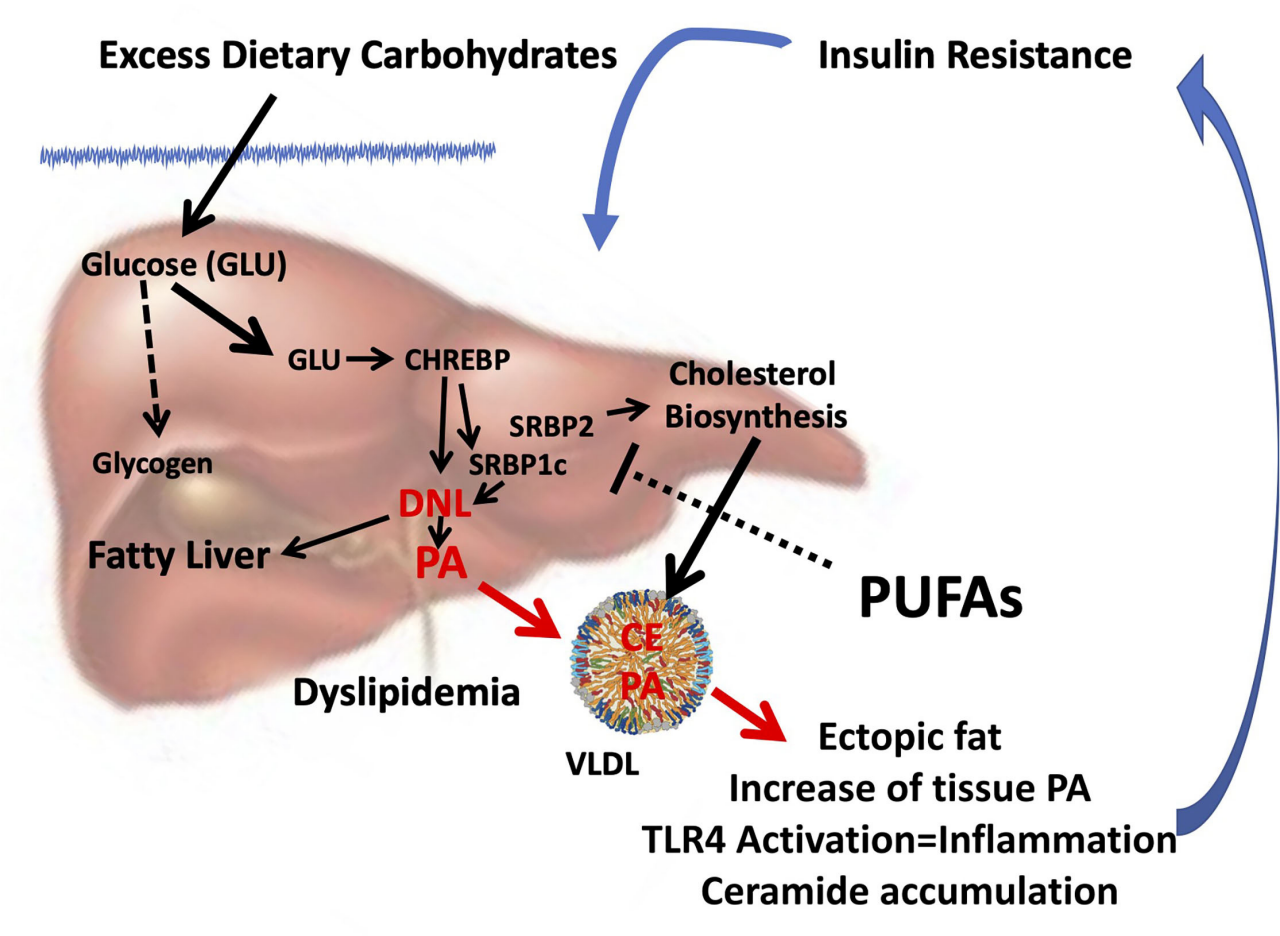
Combined consequences of liver insulin resistance and reduced PUFA intake. Insulin resistance in the liver is characterized by hyperinsulinemia and a reduced ability to store glycogen. In the presence of excess glucose, CHREBP is activated which, in turn, together with hyperinsulinemia, induces SREBP1c, and synergistically induces DNL (124) and thereby the biosynthesis of endogenous PA. Reduced PUFA intake can further promote PA and cholesterol biosynthesis since PUFAs inhibit both SREBP1c (123) and SREBP2 (125). Enhanced DNL can cause fatty liver and formation and release of VLDL enriched with PA and cholesterol esters. As a result, the accumulation of ectopic fat occurs in different tissues, and the increase in tissue PA can sustain insulin resistance by inducing inflammation through the activation of TLR4 (105) and accumulation of ceramides (106), setting in motion a vicious circle. Because reduced PUFA intake is often associated with an unbalanced increase in dietary SFA/PUFA, the rise in tissue PA can be mistakenly attributed to its dietary intake. CHREBP, carbohydrate-responsive element-binding protein; SREBP, sterol regulatory element-binding protein; DNL, *de novo* lipogenesis; PA, palmitic acid; PUFA, polyunsaturated fatty acid; TLR4, toll-like receptor 4; SFA, saturated fatty acid.

From several studies exploring the molecular mechanism of dietary FA interactions emerged that the reduction of PUFA intake, especially n-3 PUFA, rather than the excess of dietary SFA, may favor insulin resistance (123), promoting endogenous PA production *via* DNL. Thus, the increase in tissue PA from dysregulated DNL and its deleterious consequences can be mistakenly attributed to dietary intake of PA (Figure 1).

Interestingly, it has been proposed that the claimed adverse effect on cholesterol exerted by high dietary SFA/PUFA ratio may represent a physiological mechanism aimed at fulfilling the needs of tissues for cholesterol (126) and the yield of larger LDL makes this increase not to be related to CVD (7).

Many of the purported harmful effects of dietary PA are based on experimental animal studies, mainly on mice on a high-fat diet, which consists of 45–60%en whereas the optimal fat content in the rodents diets ranges from 9 to 16%en (127). Therefore, high-fat diets contain from 3- to 6-folds of the fat content required, with usually an extremely high percentage of PA and low in PUFA and n-3/n-6 PUFA ratio, which makes difficult to pinpoint the effects of a high-fat content, high concentration of PA, or high n-6/n-3 PUFA ratio. These diets were created to induce obesity as quickly as possible (128) and not to assess the nutritional impact of dietary FA. Therefore, whereas they might be suitable as a model of obesity, they cannot be taken into consideration for translational nutritional studies on the effects of dietary FA (128).

## Conclusions

Several pieces of evidence suggest that the nutritional impact of dietary FA is strictly related to the balance among them and with other macronutrients. Most of the studies claiming negative effect of PA rely on *in vitro* cell culture studies, incubating cells with extremely high concentrations and as a single FA without considering that dietary PA does not modify its tissue concentration, or with animal models of obesity with an extremely high-fat content not achievable by humans (128) and not specifically designed for studying dietary FA and thereby without any translatability to human conditions. More preclinical and clinical studies are needed to better discern the metabolic fate and interaction between dietary and *de novo* PA particularly in relation to PUFA intake, macronutrient balance, and pathophysiological states.

To blame a single nutrient, such as PA, widely present in our diet from several sources and with several well recognized fundamental physiological properties (129), as detrimental, suggesting that is sufficient to reduce its dietary intake for improving our health and prevent pathological states from CVD to cancer, is rather simplistic but it has a great praise probably because of the human nature to choose less time and energy consuming solutions for complex issues (130).

Thus, guidelines or recommendations to the general population to avoid or increase the intake of single nutrients, without considering the complexity of nutrient-nutrient interactions and the individual-specific nutritional response in relation to age, genetic, environmental, physiological and pathophysiological conditions, do not follow the amount of growing scientific data that suggest we should not be focusing on single nutrients but on increasing diet variability within a personalized nutritional approach.

## Author Contributions

EM, CM, GC, and SB: conception and design of the review, organized the literature search, wrote the first draft of the manuscript, and contributed to wrote sections of the manuscript. All authors contributed to manuscript revision, read, and approved the submitted version.

## Funding

This research was funded by Grants to GC and SB from the University of Cagliari: Fondo Integrativo per la Ricerca (FIR 2020).

## Conflict of Interest

The authors declare that the research was conducted in the absence of any commercial or financial relationships that could be construed as a potential conflict of interest.

## Publisher's Note

All claims expressed in this article are solely those of the authors and do not necessarily represent those of their affiliated organizations, or those of the publisher, the editors and the reviewers. Any product that may be evaluated in this article, or claim that may be made by its manufacturer, is not guaranteed or endorsed by the publisher.
